# A Thermal-Responsive Zwitterionic Polymer Gel as a Filtrate Reducer for Water-Based Drilling Fluids

**DOI:** 10.3390/gels8120832

**Published:** 2022-12-16

**Authors:** Kaihe Lv, Hongyan Du, Jinsheng Sun, Xianbin Huang, Haokun Shen

**Affiliations:** 1 Department of Petroleum Engineering, China University of Petroleum (East China), Qingdao 266580, China; 2 Key Laboratory of Unconventional Oil & Gas, Development Ministry of Education, Qingdao 266580, China

**Keywords:** water-based drilling fluid, thermal-responsive, salt-responsive, zwitterionic polymer gel, high temperature resistance

## Abstract

It is crucial to address the performance deterioration of water-based drilling fluids (WDFs) in situations of excessive salinity and high temperature while extracting deep oil and gas deposits. The focus of research in the area of drilling fluid has always been on filter reducers that are temperature and salt resistant. In this study, a copolymer gel (PAND) was synthesized using acrylamide, N-isopropyl acrylamide, and 3-dimethyl (methacryloyloxyethyl) ammonium propane sulfonate through free-radical polymerization. The copolymer gel was then studied using FTIR, NMR, TGA, and element analysis. The PAND solution demonstrated temperature and salt stimulus response characteristics on rheology because of the hydrophobic association effect of temperature-sensitive monomers and the anti-polyelectrolyte action of zwitterionic monomers. Even in conditions with high temperatures (180 °C) and high salinities (30 wt% NaCl solution), the water-based drilling fluid with 1 wt% PAND displayed exceptional rheological and filtration properties. Zeta potential and scanning electron microscopy (SEM) were used to investigate the mechanism of filtration reduction. The results indicated that PAND could enhance bentonite particle colloidal stability, prevent bentonite particle aggregation, and form a compact mud cake, all of which are crucial for reducing the filtration volume of water-based drilling fluid. The PAND exhibit excellent potential for application in deep and ultra-deep drilling engineering, and this research may offer new thoughts on the use of zwitterionic polymer gel in the development of smart water-based drilling fluid.

## 1. Introduction

With the progressive depletion of conventional oil and gas resources, the focus of oil and gas exploration and development has transferred to unconventional resources such as deep oil and gas, shale gas, and geothermal [1]. As the “blood” of drilling engineering, drilling fluid plays critical functions in the oil and gas extraction process, such as cooling and lubricating bits and drilling tools, transporting and suspending rock cuttings, and maintaining wellbore stability [2].

Water-based drilling fluid (WDF) and oil-based drilling fluid (ODF) are the two basic types of drilling fluid. The advantages of ODF include high temperature resistance, excellent lubricating performance, and prevention of the hydration swelling of formation minerals. However, the high cost and the severe toxicity of oil-based drilling fluid limit its application in the field. The WDF has become the first choice because of the low preparation cost, simple treatment and maintenance, wide source of treating agents, and easy performance control [3].

As the formation temperature increases with drilling depth, the drilling fluid additives in WDF will undergo high degradation, high-temperature cross-linking, high-temperature desorption, high-temperature dehydration, and other high-temperature destruction in the high-temperature and high-pressure environment of deep wells, resulting in poor performance of WDF [4,5]. Furthermore, salt beds are frequently drilled during drilling deep and ultra-deep wells. Since WDF is a colloidal system containing water, bentonite and various treatment agents, when the soluble salt in the salt beds is dissolved into the drilling fluid, the colloidal stability of the drilling fluid is reduced, negatively affecting the rheological and filtration properties of WDF [6]. Therefore, higher demands are placed on drilling fluid performance in deep well drilling operation, particularly in terms of high temperature and salt resistance. The most basic performance requirement for drilling fluid is excellent temperature and salt resistance.

Generally, the direct method to improve the high temperature and salt resistance of WDF is to use drilling fluid treatment agents with high temperature and salt resistance [7]. As one of the most important treatment agents in WDF, filtration reducers can be adsorbed on the surface of bentonite particles through physical and chemical actions such as hydrogen bonding and electrostatic force to form a weak gel structure of “bentonite-polymer-water” improve the rheological and filtration properties of WDF in a high-temperature environment [8]. When drilling into permeable formations where the hydrostatic pressure is higher than the formation pressure, water in drilling fluid will permeate into the formation. Filtration control is a crucial characteristic of a drilling fluid. Therefore, the development of filtration reducer with high temperature and salt resistance is an important subject that needs to be solved urgently in drilling fluid technology. Filtration reducer, including modified cellulose [9], humic acid [10], modified starch [11], resin [12], and vinyl monomer copolymer [13,14], is widely used in shallow drilling operations as a rheological modifier and filtration control agent in drilling fluid. Since the vinyl monomer copolymer filtrate reducer has the advantages of suitable high temperature resistance and adjustable performance, it has attracted a lot attention and has made certain research progress. At present, the way to improve the high temperature and salt resistance of filtration reducer is usually to introduce monomers with high temperature resistance and strong hydration ability into the acrylamide-based polymer. Wang et al. [15] synthesized a filtration reducer composed of N, N-dimethylacrylamide (DMAM), 2-acrylamido-2-methylpropane sulfonic acid (AMPS), N-vinylpyrrolidone (NVP) and sodium 4-styrenesulfonate (SSS) through free-radical polymerization, which is resistant to ultra-high temperature (240 °C). Chang et al. [16] prepared an environmentally friendly filtration reducer by grafting acrylamide (AM) and 2-acrylamido-2-methylpropanesulfonic acid (AMPS) onto nano lignosulfonate. The results showed that it could be applied at 200 °C. Zhu et al. [17] grafted acrylic acid (AA), acrylamide (AM), 2-acrylamido-2-methylpropane sulfonic acid (AMPS) onto xanthan gum (XG), and successfully synthesized a new environmentally friendly xanthan gum derivative XG-AA/AM/AMPS. The results show that the drilling fluid can maintain reasonable rheological properties at 180 °C. However, due to the polyelectrolyte effect, the polymer chain of the traditional polymer filtration reducer would collapse in the high-salinity environment, which weaken the interaction with the bentonite in WDF and make the rheology and filtration performance deteriorate [18,19]. In order to improve the hydration and dispersion ability of additives in high-salinity environment, hydrophilic groups such as carboxylic acid group (–COOH) and sulfonic acid group (–SO_3_H) are usually introduced into the molecular chain [8,20]. This can temporarily withstand salt intrusion, but they are intrinsically incompatible with salt. So, it cannot maintain long-term stability in a salt solution. It is difficult to maintain long-term performance stability in high-temperature and high-salinity environments [21].

In recent years, zwitterionic polymers (ZPs) have attracted much attention due to their suitable hydration ability, antifouling ability, and ionic response characteristic [22,23,24,25]. ZPs are a series of materials with the same number of cations and anions on their polymer chains. Sulfobetaine (SB)-based ZPs are most promising to be industrialized because it is easy to prepare SB monomers, and some of them are commercially available. ZPs have specific anti-polyelectrolyte effects, which can transform the antagonistic effect into a cooperative effect between polymer and salt. Compared with polyelectrolytes, the dispersion stability of ZPs is enhanced greatly under high-salinity conditions. Due to the salt ions that could shield the electrostatic attraction inside and between the polymer chains, the polymer chains are fully stretched, causing the ZP to maintain a high viscosity even in brine [26].

Different from the principle of increasing the relative molecular weight of polymer to improve the viscosity of polymer solution, Hourdet et al. [27,28,29] first proposed thermo-thickening polymer (TTP). They introduced side chains with low critical solution temperature (LCST) on the main chain of water-soluble polymers. Thermo-thickening polymer will behave as a water-soluble polymer at low temperatures and as a hydrophobic associating polymer at higher temperatures due to the hydrophilic to hydrophobic transition of side chains with low critical solution temperature. The interaction of hydrophobic regions of various polymer molecules results in the formation of a weak gel-like structure through van der Waals force, which increases flow resistance [30]. In other words, the viscosity of TTPs increases as temperature rises. N-alkyl-substituted acrylamide polymer is a kind of polymer with thermo-thickening property that was widely used in the fields of improving oil and gas recovery, plugging cross-linking agent and oil well cement suspending agent. However, the research results on TTP in WDF are limited. In theory, thermo-thickening polymer can be used to control or increase the viscosity of WDF.

**Scheme 1:**
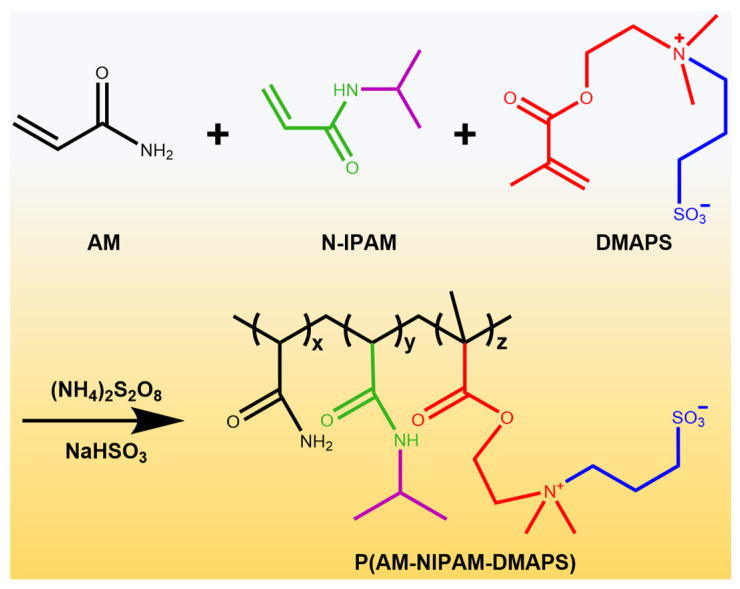
The chemical reaction formula to synthesize PAND.

Therefore, in order to combine the advantages of zwitterionic polymer and thermo-thickening polymer, a dual-responsive zwitterionic polymer gel (PAND) as a filtrate reducer for WDFs was prepared by using acrylamide (AM), N-Isopropyl acrylamide (NIPAM) and 3-dimethyl (methacryloyloxyethyl) ammonium propane sulfonate (DMAPS) via free-radical polymerization, as shown in Scheme 1. In this polymer, the introduction of thermo-sensitive monomers endows the polymer with thermo-thickening properties and improves the temperature resistance. At the same time, the introduction of zwitterionic monomers promoted the extension of molecular chains in salt water and improved the salt resistance of the polymer. Moreover, the sulfonic acid group in PAND, a hydration group, improves the colloidal stability of bentonite in drilling fluid. The water-based drilling fluid added with PAND exhibits satisfactory rheological and filtration properties even in high-temperature and high-salinity environments. This study may provide a new perspective on how to design smart water-based drilling fluid polymers with temperature and salt response characteristics.

## 2. Results and Discussion

### 2.1. Characterization of PAND

**Figure 1:**
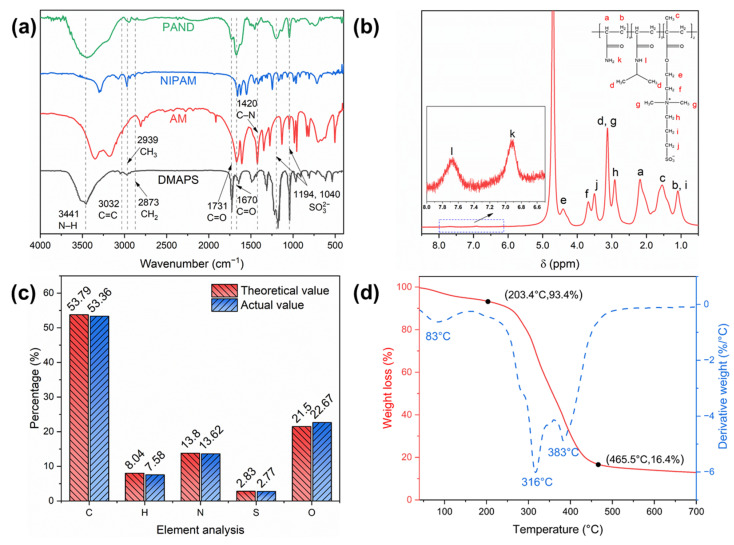
FTIR spectrum (**a**) ^1^H NMR spectrum (**b**) elemental analysis (**c**) TGA and DTG curves (**d**) of PAND.

As shown in Figure 1a, 3441 cm^−1^ is the stretching vibration peak of N-H in the amide group. A total of 2939 cm^−1^ and 2873 cm^−1^ are stretching vibration peaks of methyl and methylene, respectively. The peaks at 1670 cm^−1^ and 1420 cm^−1^ are caused by the stretching vibration of carbonyl C=O and C–N in the amide group. The peak at 1731 cm^−1^ can be attributed to the tensile vibration of the ester carbonyl group in DMAPS. The symmetric absorption peaks of sulfonic groups in DMAPS are at 1193 cm^−1^ and 1040 cm^−1^. Furthermore, the typical peaks of unsaturated olefin bonds (3032 cm^−1^) did not present in the FTIR spectrum of PAND, indicating that the reaction was complete. Furthermore, the ^1^H NMR spectrum was used to test the chemical shifts of protons in the PAND, as well as the related peaks. As shown in Figure 1b, peak a (2.18 ppm) and peak b (1.08 ppm) are proton peaks of –CH and –CH_2_ in the polymer backbone, respectively. The peak k (6.92 ppm) is attributed to the –NH_2_ in AM. The peak l (7.67 ppm) and peak d (3.12 ppm) are attributed to the –NH– and methyl in NIPAM. The peak g (3.12 ppm) should be attributed to the CH_3_–N in DMAPS. The methyl and methylene in DMAPS could be observed at 1.55 ppm (peak c), 4.39 ppm (peak e), 3.67 ppm (peak f), 2.90 ppm (peak h), 1.08 ppm (peak i), and 3.49 ppm (peak j), respectively. Moreover, as shown in Figure 1c, the elemental analysis (C, H, N, S, O) of the copolymer synthesized with the optimal monomer molar ratio of AM, NIPAM, and DMAPS (7:3:1) shows that the measured value of the element content in the copolymer was consistent with the theoretical value, indicating the successful preparation of the target product.

Excellent thermal stability is an important performance for drilling fluid treatment agents. Figure 1d showed the TG and DTG curves of PAND. According to the TG curve, the thermal decomposition of PAND can be divided into three stages: the mass loss of the first stage (40.0–203.4 °C) is 6.6%, and a peak value is displayed on the DTG curve at 83 °C. The PAND molecule contains hydrophilic groups, which is easy to absorb moisture. After the temperature rises, the adsorbed water are heated and volatilized, resulting in the mass loss in this stage. The mass loss of the second stage (203.4–465.5 °C) is 77%, and the DTG curve shows two peaks at 316 °C and 383 °C, respectively. The reason for the mass loss in this stage can be attributed to the thermal degradation of the amide group, sulfonic acid group and other side groups in PAND molecules are largely degraded, and the degradation rate is fastest at about 316 °C. The TG curve of the third stage (465.5–650 °C) decreased slowly, which was due to the mass loss caused by the gradual carbonization of the polymer. In general, PAND starts to decompose at 300 °C, which indicates that PAND has excellent thermal stability and can withstand high temperature up to 300 °C.

### 2.2. Salt Response Characteristics of the PAND Solution

**Figure 2:**
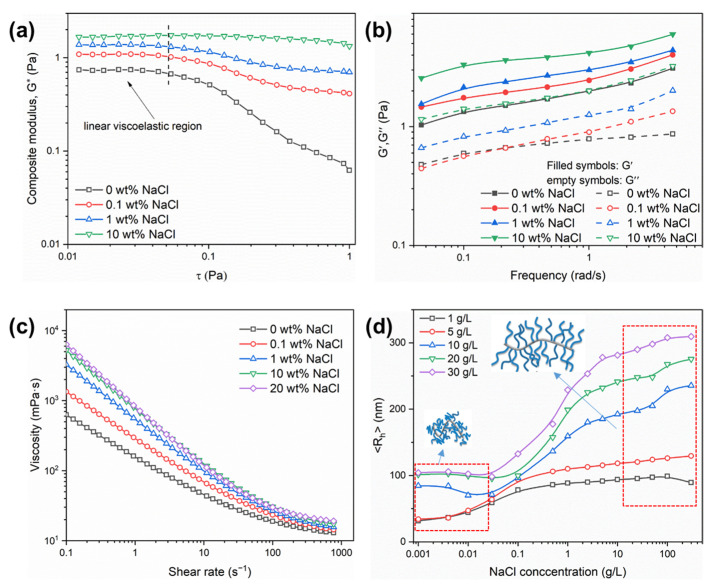
Viscoelastic behavior (**a**), the storage modulus (G′), loss storage modulus (G″) as a function of frequency (**b**), viscosity as a function of shear rate (logarithmic axes) (**c**) of 1.0 wt% PAND solution with different concentration of NaCl. (**d**) The mean hydrodynamic radius <*R_h_*> of PAND as a function of the NaCl concentration.

The most remarkable feature of zwitterionic polymer is that the molecular chain extends with the increase in ionic strength in water, which shows as the viscosity of polymer solution increases with the increase in salt concentration, macroscopically. Therefore, the rheological properties of the 1% PAND solutions with different concentrations of NaCl were analyzed by a HAAKE viscometer to reveal the salt responsiveness. Before the dynamic shear test, it is necessary to determine the linear viscoelastic zone. Figure 2a shows the curve of composite modulus (G*) of polymer solution versus stress. Within the test stress range, a linear viscoelastic zone occurs in all polymer solutions, but the specific stress range corresponding to each polymer solution is different. The ranges of linear viscoelastic region widened by the increasing NaCl concentration. The wide range of linear viscoelastic regions indicated that the entanglement or interaction between molecules in the solution system is strong. When the stress is greater than the upper limit of the stress in the linear viscoelastic region, G* starts to decrease gradually with the continuous increase in the stress, indicating that the solution system structure is damaged and shear dilution occurs. In order to make the experimental results comparable, the unified fixed stress is 0.05 Pa, and the dynamic rheological experiments were carried out. Figure 2b shows the dynamic rheological curve of 1% PAND solution under different salinity. With the increase in shear frequency, both G′ and G″ of polymer solution increase. This is because polymer solution is an aggregation of macromolecules. When the frequency increases from 0.01 rad/s to 10 rad/s, because the deformation of large aggregates under the action of external force cannot be relaxed in time, they will be transformed into elastic storage. Therefore, their elasticity improves with increasing frequency. Moreover, G′ is always higher than G″, indicating that the polymer solution shows a strong internal spatial structure and solid viscoelastic behavior [31]. In addition, with the increase in NaCl mass fraction, the G′ and G″ of polymer solution increase. Furthermore, because polymer solutions are non-Newtonian fluids, they often exhibit a shear-thinning behavior, which maintains high viscosity at low shear rates to suspend cuttings in the wellbore but low viscosity at high shear rates to be injected quickly to the bottom of the wellbore. As demonstrated in Figure 2c, the PAND solution exhibited a typical shear-thinning behavior, and the viscosity increased with the increase in NaCl contents. This phenomenon intuitively explained the salt-responsive features of the PAND solution. Salt ions can break the dipole-dipole connection within and between chains, decrease the interaction force between chains, and cause polymer chains to change from collapse to stretching conformation. The change in molecular chain conformation can be characterized by the mean hydrodynamic radius (<*R_h_*>). The effects of ionic strength on the <*R_h_*> of PAND were measured by dynamic light scattering, and the results are shown in Figure 2d. In a salt-free environment, the <*R_h_*> of PAND increases from 40 nm to 100 nm with the increase in polymer concentration. This is because when the polymer concentration is lower than the overlapping concentration (10 g/L), the anions and cations in the polymer molecular chain associate with each other through electrostatic interaction, which will cause the polymer to curl up and reduce the <*R_h_*>. When the concentration of PAND is greater than the overlapping concentration, besides the anion and cation association within the molecular chain, the electrostatic interaction between the molecular chains cannot be ignored. Electrostatic interaction between molecular chains will cause multiple polymer chains to associate together and form larger aggregates, resulting in an increase in <*R_h_*>.

When the NaCl concentration is increased to 100 g/L, the <*R_h_*> of PAND increases several times. The Debye formula, expressed as follows, can perfectly explain the above phenomenon:

$$l_{D}={\left[4\pi l_{B}C_{S}\right]}^{-1/2}$$

where $l_{B}$ is the Bjerrum length in pure water, $C_{S}$ is the concentration of salt, $l_{D}$ is the Debye length. The Debye formula represents the range of electrostatic interactions, which is the critical distance that different charges that could affect each other. When the concentration of the salt solution increases, the Debye length decreases. When the mass fraction of NaCl increases, the dipole-dipole interactions and electrostatic interaction between cations and anions in the polymer chain is shielded, and the polymer molecular chain gradually extends and forms a spatial network structure, causing the viscosity of the polymer solution to increase [32,33].

### 2.3. Temperature Response Characteristics of the PAND Solution

**Figure 3:**
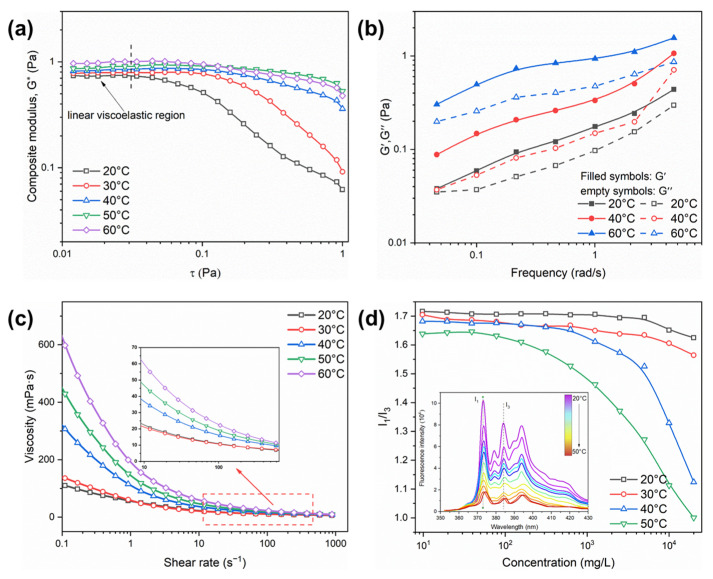
Viscoelastic behavior (**a**), the storage modulus (G′), loss storage modulus (G″) as a function of frequency (**b**), viscosity as a function of shear rate (linear axes) (**c**) of 1.0 wt% PAND solution at different temperature. (**d**) The I1/I3 curve as a function of PAND concentration at different temperatures.

The PAND solution’s temperature responsiveness was investigated using the same methodology. Firstly, the linear viscoelastic zone was determined. The composite modulus (G*) curve of the PAND solution at different temperatures is depicted in Figure 3a. In order to make the experimental results comparable, the unified fixed stress is 0.03 Pa. Figure 3b shows the change in G′ and G″ as a function of frequency at various temperatures. In general, for PAND solutions, G′ was higher than G″, indicating that PAND solutions have solid viscoelastic behavior and stable internal spatial structure. Additionally, the rise in temperature was correlated with a rise in the elastic modulus of PAND solutions. As shown in Figure 3c, the PAND solution displayed shear-thinning behavior, and the viscosity of the PAND solution increased significantly with the increase in temperature. The rheological test adequately reflected the temperature-responsive characteristics of PAND. To understand more about the molecular basis of the thermo-thickening mechanism, the fluorescent spectroscopy method was used to examine the polarity changes in the PAND solution brought about by heating. The fluorescence emission spectra of pyrene, as shown in Figure 3d, displayed the characteristic five emission peaks in sequence. The intensity ratio (I1/I3) of the first emission peak (372 nm) to the third emission peak (383 nm) is affected by the polarity of the environment in which the pyrene molecule is placed. The pyrene molecule is a hydrophobic non-polar molecule that is preferentially soluble in the hydrophobic phase of hydrophobic associative polymers. The less the I1/I3 value, the larger the volume of the hydrophobic associative structure and the less polarized the hydrophobic micro area. For the PAND solutions at 20 °C, the I_1_/I_3_ decreased from 1.72 to 1.63. However, the I1/I3 of the PAND solutions at 50 °C decreased from 1.64 to 1.01. The significant decrease in I_1_/I_3_ indicates that a large number of hydrophobic regions generate in the PAND solution. Due to the fact that the hydrogen bonds between NIPAM’s isopropyl and water molecules were broken as temperature increased, changing from a hydrophilic to a hydrophobic state above the lower critical solution temperature (LCST). The formation of strong hydrophobic interaction allows polymer chains to aggregate together, resulting in a strong 3D network structure between the polymer molecular chains and improved strength of the internal spatial structure of the PAND solution [34,35,36,37].

### 2.4. Applied in Water-Based Drilling Fluid

#### 2.4.1. Rheology and Filtration Performance

**Figure 4:**
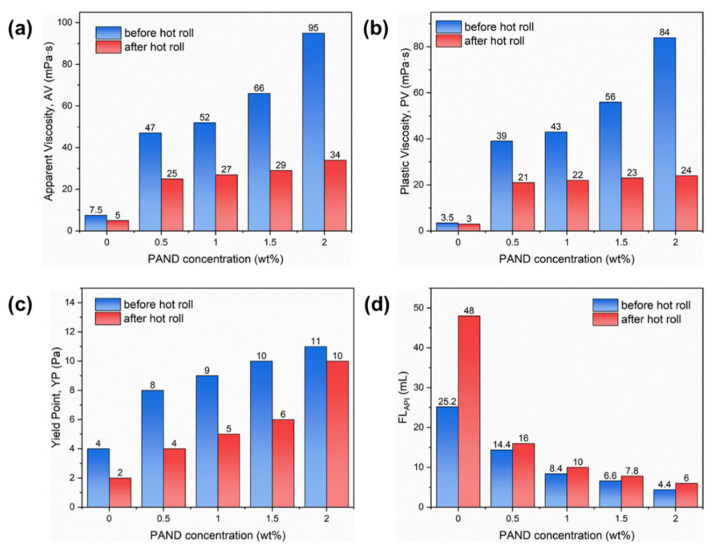
Rheological and fluid loss properties of drilling fluid with different contents of PAND before and after aging at 180 °C for 16 h: (**a**) apparent viscosity (AV); (**b**) plastic viscosity (PV); (**c**) yield point (YP); (**d**) fluid loss volume (FL_API_).

The rheological and fluid loss properties of WDFs are crucial for carrying cuttings and sustaining the wellbore stability. The apparent viscosity (AV) reflects the flowability of WDFs. The plastic viscosity (PV) reflects the friction of various materials in drilling fluid. Yield point (YP) is a function of electrochemical behavior or effect of long-chain polymers. A proper YP value means suitable suspension performance. It can be seen from Figure 4a–c that the AV, PV, and YP of drilling fluid increased with the increase in PAND contents before the hot roll all increased. When the concentration of PDANV is 2.0%, the AV, PV, YP, and filtrate loss of drilling fluid before aging were 95 mPa·s, 84 mPa·s, and 11 Pa and 4.4 mL, respectively. After aging at 180 °C, the filtrate loss is only 6.0 mL, which indicates that the PAND still has a suitable effect on reducing filtrate loss even under high temperatures. This can be attributable to the fact that PAND is adsorbed on the surface of bentonite particles under the action of electrostatic adsorption and hydrogen bonds, forming the spatial network structure of clay particles and polymer macromolecules. The bridging effect of molecular chains makes bentonite particles difficult to coalesce and maintains a certain dispersion in the drilling fluid, forming fine bentonite particles and forming a compact filter cake, reducing drilling fluid filtration. After high-temperature aging, PAND molecular chain is not easy to be degraded at high temperatures due to the strong rigidity of the molecular chain. In addition, the amide group has strong adsorption on bentonite particles, which makes bentonite particles maintain well dispersion even under high-temperature environments.

**Figure 5:**
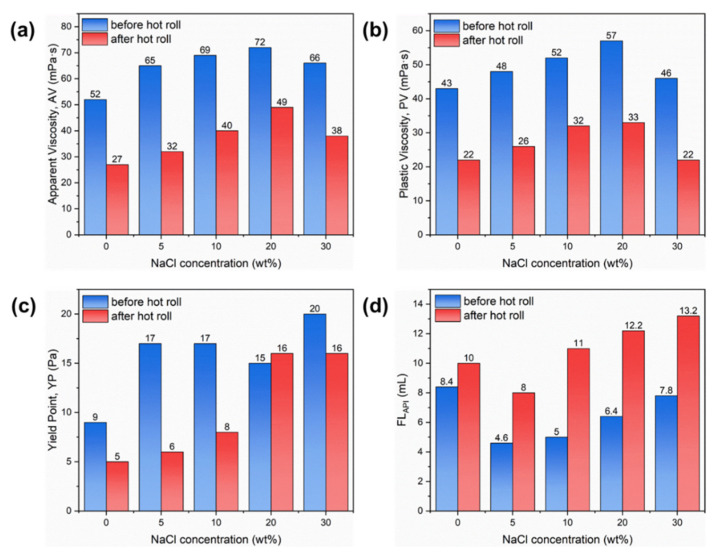
Rheological and fluid loss properties of 1 wt% PAND-based drilling fluid with different contents of NaCl before and after aging at 180 °C for 16 h: (**a**) apparent viscosity (AV); (**b**) plastic viscosity (PV); (**c**) yield point (YP); (**d**) fluid loss volume (FL_API_).

Electrolytes also affect the rheological and fluid loss properties of drilling fluids. Figure 5a–c expresses the effect of NaCl on the rheology of PAND base WDF. After adding 5 wt% NaCl, the AV increased from 52 to 65 mPa·s, the PV from 43 to 48 mPa·s, and the YP increased from 9 to 17 Pa. Moreover, when the concentration of NaCl increased to 20 wt%, the rheological parameters of the drilling fluid still increased. Due to the anti-polyelectrolyte effect of PAND, the drilling fluid can maintain relatively high viscosity and yield point under conditions of high salinity. Although the filtration loss increased with the increase in salt concentration, the increased level is limited, which can meet the requirements of drilling engineering for filtration loss.

#### 2.4.2. Filter Cake Quality

**Figure 6:**
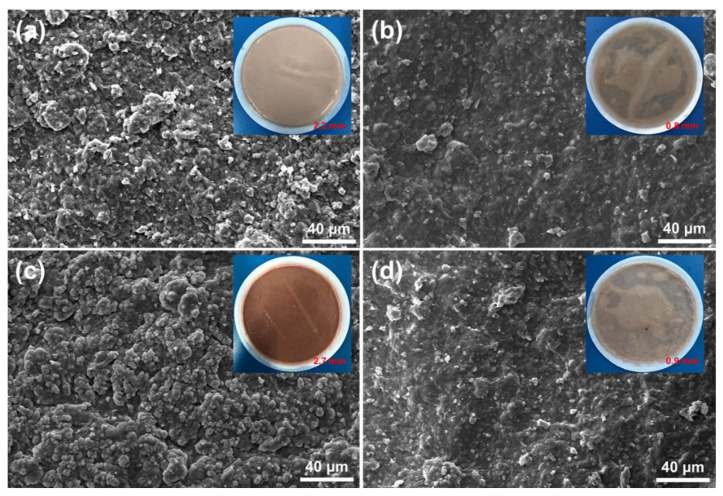
SEM and digital images of mud cakes deposited from different drilling fluids after aged at 180 °C for 16 h: (**a**) 4% bentonite WDF, (**b**) 4% bentonite + 1 wt% PAND WDF, (**c**) 4% bentonite + 30 wt% NaCl WDF, (**d**) 4% bentonite + 1 wt% PAND + 30 wt% NaCl WDF.

The filter cake is the solid sediment deposited on the well wall, ground surface, or filter paper during the filtration of drilling fluid. The quality of mud cake not only directly affects the filtration of the drilling fluid system but also has a close relationship with wellbore stability, reservoir protection, and cementing quality. Therefore, improving the quality of filter cake is of great practical significance in drilling engineering. The too-thick filter cake is easy to cause differential pressure sticking. Generally, the filter cake of drilling fluid needs to be thin, dense, and flexible. As shown in Figure 6a–d, the filter cake thickness of freshwater drilling fluid and brine drilling fluid is 2.2 mm and 2.7 mm, respectively. After adding 1 wt% PAND, the filter cake thickness of freshwater drilling fluid and brine drilling fluid is reduced to 0.8 mm and 0.9 mm, respectively. Generally, the greater the filtration loss, the greater the thickness of the filter cake. This is consistent with the above trend of fluid loss. This indicated that PAND could improve the quality of filter cake. SEM was used to further confirm the effect of PAND. The bentonite particles of freshwater drilling fluid are large. The bentonite particles of brine drilling fluid are obviously agglomerated, and there are many holes can be observed. After adding 1 wt% PAND, the filter cake surface becomes relatively smooth, without large clay particles and obvious holes and cracks, which indicates that the addition of PAND makes the clay disperse evenly and stack tightly, preventing the agglomeration of bentonite particles under the conditions of high temperature and high salinity, thus improving the compactness of the filter cake.

#### 2.4.3. Colloidal Stability Analysis

**Figure 7:**
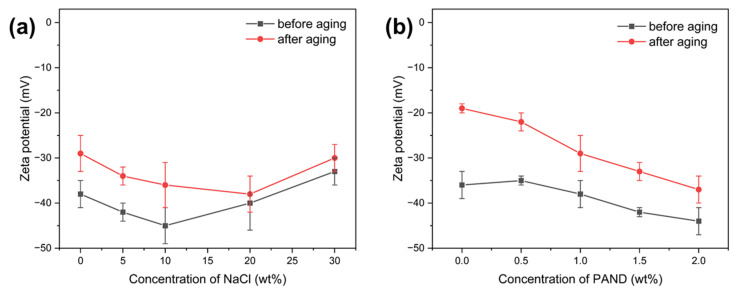
(**a**) Zeta potential curves of 4% bentonite WDF with different contents of PAND before and after aging. (**b**) Zeta potential curves of 1 wt% PAND drilling fluids with different fractions of NaCl before and after aging. The aging temperature is 180 °C.

Drilling fluid is a colloidal dispersion system composed of bentonite particles, polymer, and water. According to the DLVO theory, there are attractive and repulsive forces between particles. When particles collide with each other in Brownian motion, if the attractive force is greater than the repulsive force, particles will coalesce. On the contrary, particles maintain their stability. As one of the most important repulsive forces between particles, the electrostatic repulsive force between particles is much smaller than the zeta potential. Therefore, studying the change of zeta potential can reflect the stability of drilling fluid colloids. Figure 7 displays the results of the zeta potential of WDFs before and after aging. As illustrated in Figure 7a, the absolute value of the zeta potential of freshwater drilling fluid gradually rises both before and after aging with an increase in PAND concentration. The zeta potential of freshwater drilling fluid without PAND before aging is similar to the potential of freshwater drilling fluid with PAND after high-temperature aging when the mass fraction of PAND increases to 2%, indicating that PAND could improve the colloid stability of drilling fluid [38]. Additionally, as shown in Figure 7b, the zeta potential of the drilling fluid exhibits a tendency of first increasing and then reducing in the saline drilling fluid containing 1 wt% PAND as the concentration of NaCl gradually increases. Due to the large amount of anionic sulfonic acid groups and the ability of inorganic salts to facilitate the dissolution of PAND molecular chains in water, the diffusion double electric layer of bentonite particles is thicker, and the absolute value of zeta potential is larger. When the concentration of NaCl is 30 wt%, the zeta potential of drilling fluid after aging is still higher than 30 mV, indicating that the drilling fluid maintains well colloidal stability. Consequently, the above results both demonstrate that the PAND can effectively maintain the colloidal stability of bentonite particles in high-temperature, high-salinity environments.

## 3. Conclusions

In this work, a dual-responsive (temperature- and salt-responsive) zwitterionic polymer gel (PAND) was synthesized using AM, NIPAM, and DMAPS via free-radical copolymerization as a filtrate reducer in the water-based drilling fluid. The result of TGA demonstrated that the PAND exhibited excellent thermal stability. Rheological experiments show that the PAND solution exhibited thermal-thickening and salt-thickening properties. This is because the temperature-sensitive monomer in PAND changes from hydrophilic to hydrophobic after heating, and hydrophobic association occurs, which enhances the interaction between molecular chains and increases the viscosity of the solution. Furthermore, due to the anti-polyelectrolyte effect of zwitterionic monomer, the conformation of PAND gradually expands as the ionic strength of the solution increases, making the PAND solution exhibit a unique salt-thickening property. Even after aging at 180 °C, the drilling fluids displayed exceptional rheological and filtration properties (FL_API_ < 10 mL) when 1 wt% PAND was added to the freshwater drilling fluid and brine drilling fluid. The results of zeta potential and SEM showed that the PAND could increase the colloidal stability of WDF in a high temperature and high saline environment and may promote the formation of a thin and compact filter cake, which can greatly minimize the filtration of the drilling fluid. This research may offer fresh ideas on how to design polymers with high temperature- and salt-resistance properties for application in WDFs under circumstances of extremely high temperature and high salinity.

## 4. Materials and Methods

### 4.1. Materials

Acrylamide (AM), N-Isopropyl acrylamide (NIPAM), and 2-(Dimethylamino) ethyl methacrylate (DMAEMA) were purchased from Aladdin Industrial Co., Ltd. (Shanghai, China). Acetone, sodium hydroxide (NaOH), ammonium persulfate ((NH_4_)_2_S_2_O_8_), sodium bisulfite (NaHSO_3_), and sodium chloride (NaCl) were purchased from Sinopharm Chemical Reagent Co., Ltd. (Shanghai, China). Sodium-based bentonite (Na-bt) was obtained from J&K Scientific Ltd. (Shanghai, China).

### 4.2. Synthesis and Characterization of P(AM-NIPAM-DMAPS) Copolymer

Firstly, 5.2 g AM, 7.2 g NIPAM, and 12.4 g DMAPS were dissolved into 100 mL deionized water with stirring at room temperature. Next, the pH of the solution was adjusted to 7 using a 20 wt% NaOH solution. The solution was transferred to a three-port flask and protected by nitrogen for 30 min. Then, 50 mg (NH_4_)_2_S_2_O_8_ and 25 mg NaHSO_3_ were added, and the solution was heated up to 58 °C for 6 h. After the reaction, the mixture was washed with absolute ethanol and acetone three times. Finally, the PAND was obtained by drying and grinding.

### 4.3. Characterization Methods of PAND

The structure of the PAND was characterized by FTIR and ^1^H NMR. The KBr pellets method was used to obtain the FTIR spectra. Using an IRTracer-100 FTIR spectrometer (Shimadzu, Kyoto, Japan), the FTIR of the materials was recorded in the 4000–400 cm^−1^. A Brucker Advance DPX-300 spectrometer (Brucker, Mannheim, Germany) was used to record the ^1^H NMR spectra of the PAND with a 30° pulse at 25 °C. For the measurement, 5 mg of samples were dissolved in 0.7 g of water and placed into an NMR tube. For better dissolution, a little NaCl was added into D_2_O. The elemental composition of PAND was analyzed by an Elementar Vario EL instrument. On a Mettler Toledo TGA/SDTA 851 equipment, thermogravimetric analysis (TGA) was carried out over a temperature range of 40–650 °C at a heating rate of 10 K/min under a N_2_ atmosphere. 

### 4.4. Preparation of PAND Solution

The PAND was dissolved in deionized water by stirring, getting a PAND solution at 20 g/L. Then, the solution was diluted with water to the given concentration. The salinity was adjusted by adding the corresponding weight of NaCl.

### 4.5. Rheological Properties of Polymer Solution

A cone-and-plate geometry rheometer with a HAAKE Mars III (Thermo Scientific, Waltham, MA, USA) rheometer (diameter 60 mm, angle 1°) was used to measure the rheological properties. With shear stress ranging from 0.01 to 1 Pa, the complex modulus (G*) of the PAND solution and drilling fluid based on ASML was also determined. The elastic modulus (G′) and loss modulus (G″) of PAND solutions were also tested in the 0.01 to 5 rad/s range for angular velocity variation. The PAND solution’s viscosity was determined using a shear rate range of 1 to 1000 s^−1^ and a frequency of 1 Hz.

### 4.6. Pyrene Fluorescence Probe

Shimadzu RF-5301PC fluorescence spectrometer was used with a fixed excitation and emission slit width of 5 nm and a spectral range of 350 to 580 nm, and the scanning speed was 300 nm/min. Pyrene was used as a fluorescence probe to measure the hydrophobicity of the PAND solution at different temperatures. Use the saturated aqueous solution of pyrene with a concentration of about 3 × 10^−5^ mol/L to prepare copolymer solutions of various concentrations. The fluorescence emission spectrum of pyrene solution has five fluorescence peaks near 373 nm, 379 nm, 384 nm, 394 nm, and 480 nm, respectively. Calculate the intensity ratio of the first peak (373 nm, I_1_) and the third peak (384 nm, I_3_) in the fluorescence spectrum.

Dissolve 10 mg of pyrene in about 800 mL of distilled water, and treat it with an ultrasonic water bath for 2 h until pyrene is completely dissolved; then, transfer the solution to a 1000 mL volumetric flask and fix the volume with distilled water to obtain a saturated pyrene solution.

### 4.7. The Mean Hydrodynamic Radius

Using a Malvern Mastersizer 3000 particle size analyzer, the mean hydrodynamic radius of PAND was measured (Malvern, U.K.). Measurements were performed at 25 °C with disposable sample cells and a 90° detection angle. The concentration of all samples was diluted to around 10.0 g/L with deionized water.

Dynamic light scattering (DLS) measures the fluctuation in intensity of scattered light that occurs due to the random movements of particles in solution (Brownian motion). The intensity of scattered light fluctuates because the particles are constantly moving. The intensity fluctuates slower for large particles due to their slower diffusion rate and fluctuates faster for smaller particles that diffuse quickly through the solution. Thus, by measuring the velocity of fluctuations, the diffusion coefficient of the particles can be determined. The particle size can then be calculated using the Stokes–Einstein equation:

$$R_{h}=\frac{kT}{6\pi \varphi_{s}D}$$

where $R_{h}$ is the hydrodynamic radius, k is the Boltzmann constant, T is the temperature in Kelvin, $\varphi_{s}$ is the viscosity of the solvent, and D is the diffusion coefficient.

DLS assumes that the particles in the solution are perfect hard spheres. However, we know that polymers in suitable solvents are shaped like expanded coils. Thus, the radius calculated from DLS corresponds to the apparent size of the dissolved particle or the hydrodynamic radius. The hydrodynamic radius is directly proportional to the rms end-to-end distance:

$$R_{h}=const.{\langle R^{2}\rangle }^{1/2}$$

### 4.8. Preparation of Water-Based Drilling Fluids

The sodium-based bentonite (16.0 g) and 1.5 g of Na_2_CO_3_ were mixed with 400.0 g of fresh water with stirring to prepare the freshwater drilling fluid and then pre-hydrated for 24 h at room temperature. The brine drilling fluid was prepared by adding various mass fractions of NaCl into the freshwater drilling fluid. A specified amount of PAND was dissolved in 400 mL of freshwater drilling fluid and brine drilling fluid with 5000 rpm stirring for 20 min in order to evaluate the impact of PAND on the performance of the drilling fluid.

### 4.9. Rheological Properties of WDFs

According to American Petroleum Institute (API) standards, a six-speed rotating viscometer (ZNN-D6; Qingdao Haitongda, China) was used to measure the viscosity of WDF. At different rotation speeds of 600 and 300 rpm, the viscometer’s reading value (θ_600_, θ_300_) was noted. Calculations of the apparent viscosity (AV), plastic viscosity (PV), and yield point (YP) were performed using Equations (1)–(3).

(1) $$\mathrm{AV}=\frac{\theta_{600}}{2}\left(\mathrm{mPa}\cdot s\right)$$

(2) $$\mathrm{PV}=\theta_{600}-\theta_{300}\left(\mathrm{mPa}\cdot s\right)$$

(3) $$\mathrm{YP}=0.5\left(\theta_{300}-\mathrm{PV}\right)\left(\mathrm{mPa}\cdot s\right)$$

### 4.10. Filtration Properties of WDFs

The filtration test procedure was carried out according to the American Petroleum Institute drilling fluid laboratory test standard. API filtration loss (FL_API_) of WDF before and after aging was evaluated by using a low-pressure filter (SD3; Tongchun, Qingdao, China). Collected and recorded the filtrate volume for 30 min at 0.69 MPa. The WDF was aged for 16 h in a hot rolling furnace (BGRL-5; Qingdao Tongchun) to evaluate its temperature resistance.

### 4.11. Zeta Potential

A nanoparticle potentiometer (Zetasizer Nano Z, Malvern Instruments Co., Ltd., Malvern, U.K.) was used to measure the zeta potential value of WDF samples.

### 4.12. Microscopic Morphology of Filter Cake

After the filtration test, the thickness of the filter cake was tested by an automatic tester for the thickness of filter cake (ZN-1L, Qingdao Senxin Group Co., LTD, Qingdao, China) and was lyophilized to constant weight. The micromorphologies of the surface of filter cakes were observed using an EVO-15 SEM (ZEISS, Aalen, Germany) at an accelerating voltage of 20 kV. Before observation, the samples were gold coated using a sputter coater.

## Data Availability

Not applicable.
